# Identification and characterization of long noncoding RNAs involved in the aluminum stress response in *Medicago truncatula via* genome-wide analysis

**DOI:** 10.3389/fpls.2022.1017869

**Published:** 2022-09-23

**Authors:** Qihui Gui, Zhengyu Yang, Chao Chen, Feng Yang, Song Wang, Rui Dong

**Affiliations:** ^1^Department of Grassland Science, College of Animal Science, Guizhou University, Guiyang, China; ^2^Key Laboratory of Animal Genetics, Breeding and Reproduction in the Plateau Mountainous Region, Ministry of Education, Guizhou University, Guiyang, China; ^3^Guizhou Technological College of Machinery and Electricity, Duyun, China; ^4^Grassland Technology Experiment and Extension Station, Guiyang, China

**Keywords:** long noncoding RNA, *Medicago truncatula*, aluminum stress, molecular regulatory mechanism, high-throughput sequencing

## Abstract

Numerous studies have shown that plant long noncoding RNAs (lncRNAs) play an important regulatory role in the plant response to environmental stress. However, there are no reports on lncRNAs regulating and enhancing aluminum (Al) stress tolerance in legumes. This study analyzed the role of lncRNAs in response to Al stress in the legume model plant *Medicago truncatula*. A total of 219.49 Gb clean data were generated: 3,284 lncRNA genes were identified, of which 515 were differentially expressed, and 1,254 new genes were functionally annotated through database alignment. We further predicted and classified putative targets of these lncRNAs and found that they were enriched in biological processes and metabolic pathways such as plant hormone signal transduction, cell wall modification and the tricarboxylic acid (TCA) cycle. Finally, we characterized the functions of 2 Al-activated-malate-transporter-related lncRNAs in yeast. The recombinant plasmids of MSTRG.12506.5 and MSTRG.34338.20 were transformed into yeast, and these yeast exhibited better growth than those carrying empty vectors on medium supplemented with 10 μM AlCl_3_ and showed that they have biological functions affording Al stress tolerance. These findings suggest that lncRNAs are involved in regulating plant responses to Al stress. Our findings help to understand the role of lncRNAs in the response to Al stress in legumes and provide candidate lncRNAs for further studies.

## Introduction

Aluminum (Al) is one of the most abundant metal elements in the earth’s crust and is deprotonated to Al^3+^ in acidic soils with a pH < 5.0, while at pH > 5.0, Al usually exists in soil in the form of poorly soluble aluminosilicates or alumina that are nontoxic to plants (Kochian et al., 2004). Micromolar concentrations of Al^3+^ in the soil can inhibit plant root growth in a short period of time and cause the rapid accumulation of reactive oxygen species (ROS), hydrogen peroxide (H_2_O_2_) and hydroxyl radicals (-OH), resulting in Al toxicity (Poot-Poot and Hernandez-Sotomayor 2011). Approximately 40% of the world’s arable land is acidic, and Al toxicity is considered one of the main factors limiting crop yields in acidic soil (Liu et al., 1995; Von Uexküll and Mutert 1995). Previous studies found that Al stress caused severe damage to the structure and function of mitochondrial membranes in peanut (*Arachis hypogaea*), while increased mitochondrial antioxidant system activity reduced cellular damages under Al stress (Zhan et al., 2014). Many plant species have evolved complex mechanisms to cope with Al toxicity; one is the exclusion mechanism in which Al is prevented from entering the root tips (symplast and apoplast), and the other is internal tolerance, which depends on detoxification and sequestration of Al in apoplastic spaces (Lou et al., 2016). Among these mechanisms, the most important one is the apoplastic exclusion strategy, which is based on root-secreted organic acids that chelate Al. Some plants can produce complexes that chelate with Al ions in the soil through the secretion of organic acids such as malic acid, citric acid and oxalic acid from their roots, thereby reducing the effects of Al toxicity (Dissanayaka et al., 2021).

In the model plant species *Arabidopsis thaliana*, *ALUMINUM-ACTIVATED-MALATE-TRANSPORTER 1* (*AtALMT1*) was identified as a key gene for Al tolerance, and root malate secretion was shown to be regulated by the transcription factor SENSITIVE-TO-PROTON-RHIZOTOXICITY 1 (STOP1), which mediates Al-induced expression of *AtALMT1* and is critical for Al resistance of *A. thaliana* (Tokizawa et al., 2021). Studies have shown that the transcription factor WRKY47 regulates the expression of *EXTENSIN-LIKE PROTEIN* (*ELP*) and *XYLOGLUCAN ENDOTRANSGLUCOSYLASE-HYDROLASE 17* (*XTH17*), which are responsible for cell wall modification, thereby balancing the Al distribution between the apoplast and symplast in the roots (Li et al., 2020).

Noncoding RNAs (ncRNAs) longer than 200 nucleotides are called long noncoding RNAs (lncRNAs), have relatively low protein-coding capacity and compose the largest class of ncRNAs (Wang et al., 2017; Yu et al., 2022). LncRNAs lack distinct open reading frames (ORFs), are mainly transcribed by RNA polymerase II, and exhibit tissue- and cell-specific expression patterns (Wang et al., 2015; Zhao et al., 2020). Based on their relative positions to protein-coding genes and genomic origin, lncRNAs can be further classified into lincRNAs, antisense-lncRNAs, intronic-lncRNAs, and sense-lncRNAs (Ponting et al., 2009). In plants, a large number of lncRNAs involved in the regulation of root development (Chen et al., 2018), flowering (Csorba et al., 2014), fruit ripening (Zhu et al., 2015), and responses to biotic (Cui et al., 2020) and abiotic stresses (Wang et al., 2017) have been characterized. Studies have shown that the bra-miR172a-lncRNA interaction is involved in the regulation of target genes associated with heat tolerance in Chinese cabbage (*Brassica rapa* ssp. chinensis) (Wang et al., 2019). Yu et al. (2022) found that lncRNAs play a role in the senescence of *Medicago truncatula* root nodules on the basis of their effects on the transport of transmembrane substances. Zhao et al. (2020) identified a set of lncRNAs responsive to cold treatment in *M. truncatula* seedlings and further analyzed the potential regulatory network of an CBF intergenic lncRNA (*MtCIR1*) and *MtCBF*, which play key roles in the cold stress response. However, lncRNA studies related to Al stress are rare.

To identify the lncRNAs corresponding to Al stress in *M. truncatula*, we performed high-throughput strand-specific RNA sequencing (RNA-seq) on root tip tissue under Al stress to study and characterize lncRNAs associated with the response to Al stress. Our findings provide new insights into the potential functions of lncRNAs during Al stress.

## Materials and methods

### Plant materials and treatments

The study was carried out in the laboratory of the College of Animal Science, Guizhou University. Seeds of *M. truncatula* A17 were surface-sterilized with 1% NaClO solution for 5 min, rinsed 5 times with distilled water, and germinated in the dark at 25°C for 3 days. Then, they were cultured in Hoagland nutrient solution (pH 5.8) at constant temperature (25°C, 16 h/8 h light/darkness) for 7 days, during which the nutrient solution was changed every 2 days (Bugbee 2004). Then, 240 same growthing seedlings were separated averagely into four groups, which included three Al-treatment time point groups (4, 24, and 48 h) in a 0.5 mM CaCl_2_ and 10 μM AlCl_3_ (pH 4.5) solution and one control (0 h) group, which was cultivated for 48 h in a 0.5 mM CaCl_2_ solution (pH 4.5). To reduce the circadian rhythm effects, the seedlings of control and 48 h groups were treated at the same time, and harvested after 48 h. For 4 h and 24 h treatment groups, their seedlings began to be treated 44 h and 24 h after the treat time of 48 h treatment group, respectively, and harvested at the same time as 48 h and control groups. Each 20 plants served as a replicate with three replicates per treatment. After the treatment, 1.5-cm-long root tips were taken, immediately frozen in liquid nitrogen, and stored at −80°C.

### Physiological trait determination

Root length was measured using a digital Vernier caliper, and root activity was measured using the naphthylamine microplate method (TB1011, Beijing Leagene Biotechnology Co., Ltd., China). The malondialdehyde (MDA) content, peroxidase (POD) activity, superoxide dismutase (SOD) activity, catalase (CAT) activity, and soluble sugar (SS) content were measured using corresponding kits from Beijing Solarbio Science and Technology Co., Ltd. (MDA-BC0020, POD-BC0090, SOD-BC0170, CAT-BC0200, SS-BC0035, Solarbio, Beijing, China). Refer to manufacturer’s instructions for measurement method.

### RNA extraction, cDNA library construction and sequencing

RNA extraction, quantitative measurements and quality assessments were performed according to the methods described by Liu et al. (2016). An Epicenter Ribo-zero™ rRNA Removal Kit (Epicenter, United States) was used to remove rRNA. The resulting libraries were sequenced by staff at Biomarker Co., Ltd. (BMKcloud, Beijing, China), on an Illumina NovaSeq 6,000 platform.

Clean reads were obtained by removing the inclusion adapters and low-quality reads from the raw data (Zhao et al., 2020). The reads were subsequently mapped to the *M. truncatula* reference genome MedtrA17_4.0 using Bowtie2 and TopHat2, after which they were assembled by StringTie (Pertea et al., 2015). LncRNAs were screened using the CPC, CNCI and Pfam platforms (Kong et al., 2007; Sun et al., 2013; Wang et al., 2013). All the transcripts were greater than 200 bp in length.

### Analysis of differentially expressed lncRNAs

The DESeq R package v1.10.1 was used for lncRNA differential expression analysis (Anders and Huber 2010). LncRNAs or mRNAs for which p was <0.05 and the log2(fold-change) was ≥1.5 were considered differentially expressed (Frazee et al., 2015). TargetFinder (v1.0) was used to predict target lncRNAs of microRNAs (Fahlgren and Carrington 2010).

### Annotation and functional analysis of DElncRNA target genes

The functions of the genes were determined on the basis of BLASTX alignment of the sequences to the contents of the NCBI nonredundant (Nr) protein sequence, Gene Ontology (GO), Clusters of Orthologous Groups of proteins (COG), Kyoto Encyclopedia of Genes and Genomes (KEGG) and SwissProt Protein databases (*E*-value < 10-5) (Yu et al., 2022). The TopGO R package and KOBAS software were used for GO enrichment analysis and KEGG pathway analysis, respectively (Mao et al., 2005). GO terms with corrected *p*-values < 0.05 were taken as the significantly enriched by differentially expressed genes.

### Quantitative real-time PCR validation

Total RNA was extracted by TRIzol reagent, and random reverse primers were used for reverse transcription of lncRNAs and mRNAs. A SYBR Premix Ex Taq II Kit (TaKaRa, Dalian, China) and Power SYBR Green Master Mix (Applied Biosystems) in conjunction with an iQ 5 Multicolor Real-time PCR Detection System (Bio-Rad, USA) were used for qRT–PCR analysis, and the primers used are designed by Premier 5 (Supplementary Table S1). The relative gene expression levels were calculated using the 2^–∆∆Ct^ method (Giulietti et al., 2001).

### Al tolerance test of transgenic yeast

The full-length cDNA sequence of the genes were obtained *via* seamless cloning, the results of which are shown in Supplementary Table S2. Transformation was performed according to a method described previously (Kawai et al., 2010). pBI121 was used as an expression vector of yeast strain INVSc1 (Invitrogen, Carlsbad, USA). The Al stress tolerance was evaluated in SC-Ura media, and the yeast cells were incubated at 28°C while being shaken for 36 h. Serial dilutions were spotted onto SC-Ura agar plates supplemented with 10 μM AlCl_3_ and incubated at 28°C for 48 h, all of which was replicated 3 times.

In addition, transgenic yeast solution (1% inoculum) was inoculated into SCu/2% (w/v) glucose liquid media supplemented with 10 μM AlCl_3_, incubated at 28°C and 180 rpm for 24 h, and then analyzed spectrophotometrically to measure the OD_600_. The OD_600_ value of transgenic yeast grown in liquid media without AlCl_3_ was used as a control (Jiang et al., 2022).

## Results

### Physiological changes in seedlings under Al stress

**Figure 1:**
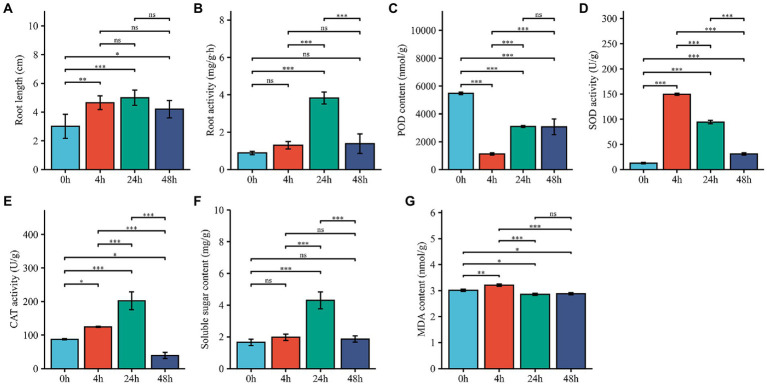
Effects of Al treatment on physiological characteristics. **(A–G)** Changes in root length, root activity, POD activity, SOD activity, CAT activity, SS content and MDA contents in root samples harvested from plants treated for 0, 4, 24, and 48 h. 0 h represents control samples, and 4, 24, and 48 h represent 4-h, 24-h, and 48-h Al stress-treated samples. The error bar represent the SDs across 3 different biological duplicates (*n* = 3). The asterisks represent significant differences in acid Al-treated samples compared with the controls (*p < 0.05; **p < 0.01; ***p < 0.001).

To investigate the Al stress tolerance of *M. truncatula* and whether the redox system plays a role in the Al stress response, we measured the root length; root activity; POD, SOD and CAT activities; and SS and MDA contents. Root length and root activity had similar trends; both began to increase after 4 h of treatment, reached their highest values at 24 h, and then showed a decreasing trend (Figures 1A,B). Compared with that at 0 h, the POD activity at 4 h decreased significantly, increased and then leveled off at 24 h (Figure 1C). The SOD activity increased significantly at 4 h and decreased from 24 h to 48 h (Figure 1D), and The CAT activity increased significantly at 4 h and 24 h and decreased rapidly at 48 h (Figure 1E). The SS content significantly increased and peaked at 24 h, after which it decreased at 48 h (Figure 1F). The MDA content increased slightly after 4 h of treatment and decreased slightly at 24 h and 48 h (Figure 1G). SOD and CAT can scavenge oxides (ROS), hydrogen peroxide (H_2_O_2_) and hydroxyl radicals (-OH) accumulated in cells. The significant increase in SOD and CAT at 4 h after *M. truncatula* exposure to Al stress suggest that cell development and cellular antioxidant-reduction systems respond rapidly to stress.

### Identification and characterization of lncRNAs

**Figure 2:**
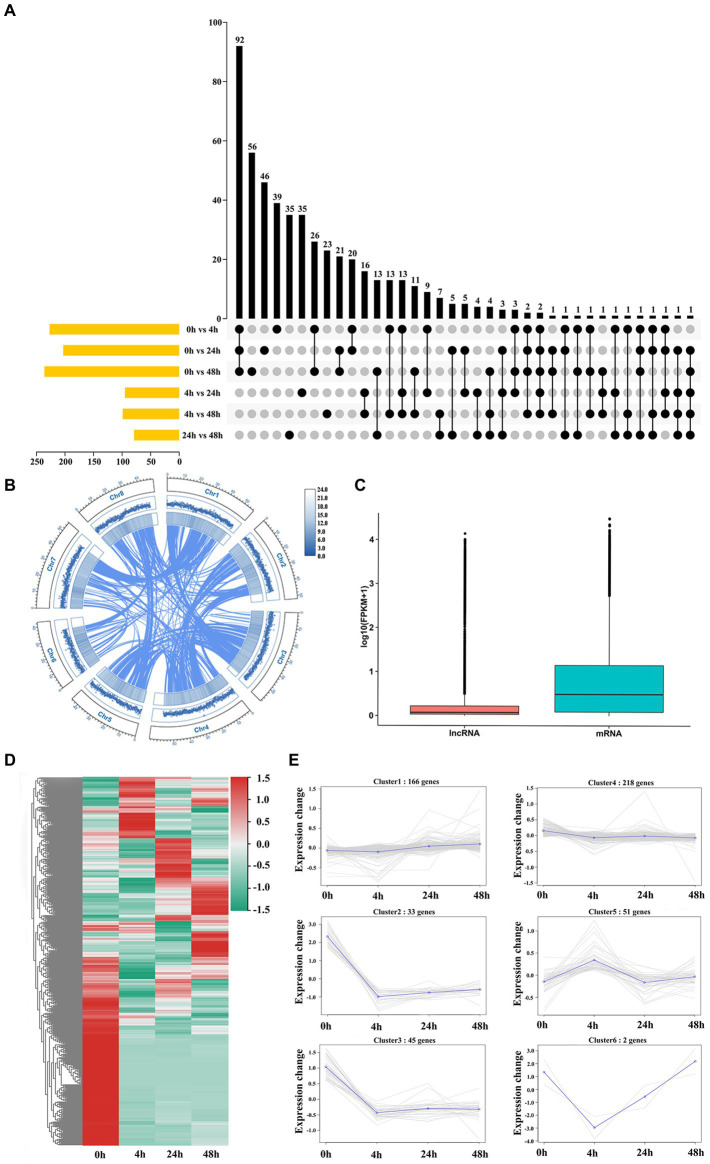
Genome-wide analysis identification and characterization of lncRNAs. **(A)** DElncRNA distribution in each group. **(B)** Circos plot of the lncRNA distribution across chromosomes. From outside to inside, lncRNA, genome feature list and linked information. **(C)** Expression levels of lncRNAs and mRNAs. **(D)** Heatmap of 515 DElncRNAs. **(E)** Coexpression cluster analysis of 515 DElncRNAs.

To identify *M. truncatula* lncRNAs involved in the Al stress response, 12 cDNA libraries were used for RNA sequencing. A total of 54,448,590 to 71,603,639 bp of clean reads were generated from each sample, with a mean GC content and number of bases of 42.65% and 1,828.97 Mb, respectively, and a mean Q30 of 94.00% (Supplementary Table S3). The average mapped reads, uniquely mapped reads and multiple-mapped reads constituted 87.70, 79.53 and 8.10% of all libraries, respectively, and the comparison efficiency of reads in each sample with the reference genome ranged from 73.13 to 93.17% (Supplementary Table S4). According to the statistics of the functional annotation results, the most new genes were annotated in the Nr and TrEMBL databases, 1,234 and 1,232, respectively (Supplementary Table S5). A total of 3,284 lncRNAs were obtained (Supplementary Figure 1A). The highest number of DElncRNAs were coexpressed at 0 h vs. 4 h, 0 h vs. 24 h and 0 h vs. 48 h (Figure 2A). Most lncRNAs were lincRNAs (2,125, 64.7%), followed by antisense (692, 21.1%), intronic (95, 2.9%) and sense (372, 11.3%) lncRNAs (Supplementary Figure 1B). The basal genomic features of 3,284 lncRNAs were characterized, the results of which revealed a uniform distribution of lncRNAs across chromosomes, with no apparent chromosomal preference (Figure 2B). The differences between lncRNAs and mRNAs were characterized by comparing the differences in transcript length, ORF length, exon number and isoform number between lncRNAs and mRNAs. The mean expression levels of mRNAs were higher than those of lncRNAs (Figure 2C). Most lncRNAs had transcript lengths <750 nt (60.38%; Supplementary Figure 1C) and ORF lengths ≤200 amino acids (aa) (Supplementary Figure 1E). The average length of mRNAs was >2,289.58 bp (Supplementary Figure 1D), and 61.38% of mRNAs had ORFs >100 aa (Supplementary Figure 1F). A total of 74.61% of lncRNAs had two exons (Supplementary Figure 1G), and 72.08% of mRNAs had more than three exons (Supplementary Figure 1H). The consistency of the boxplots for the expression of the 12 samples indicated that the gene expression level distributions of individual samples were generally consistent with a low degree of dispersion (Supplementary Figure 1I). The presence of one or both isoforms is the most common case for isoform distribution in lncRNAs and mRNAs (Supplementary Figure 1J). A total of 515 DElncRNAs were identified, and these lncRNAs clustered into 6 groups with similar expression patterns, namely, 4 groups whose members exhibited downregulated (2, 3, 4 and 6) patterns and 2 groups whose members exhibited upregulated (1 and 5) patterns (Figures 2D,E).

### Functional analysis of DElncRNAs in response to Al stress

**Figure 3:**
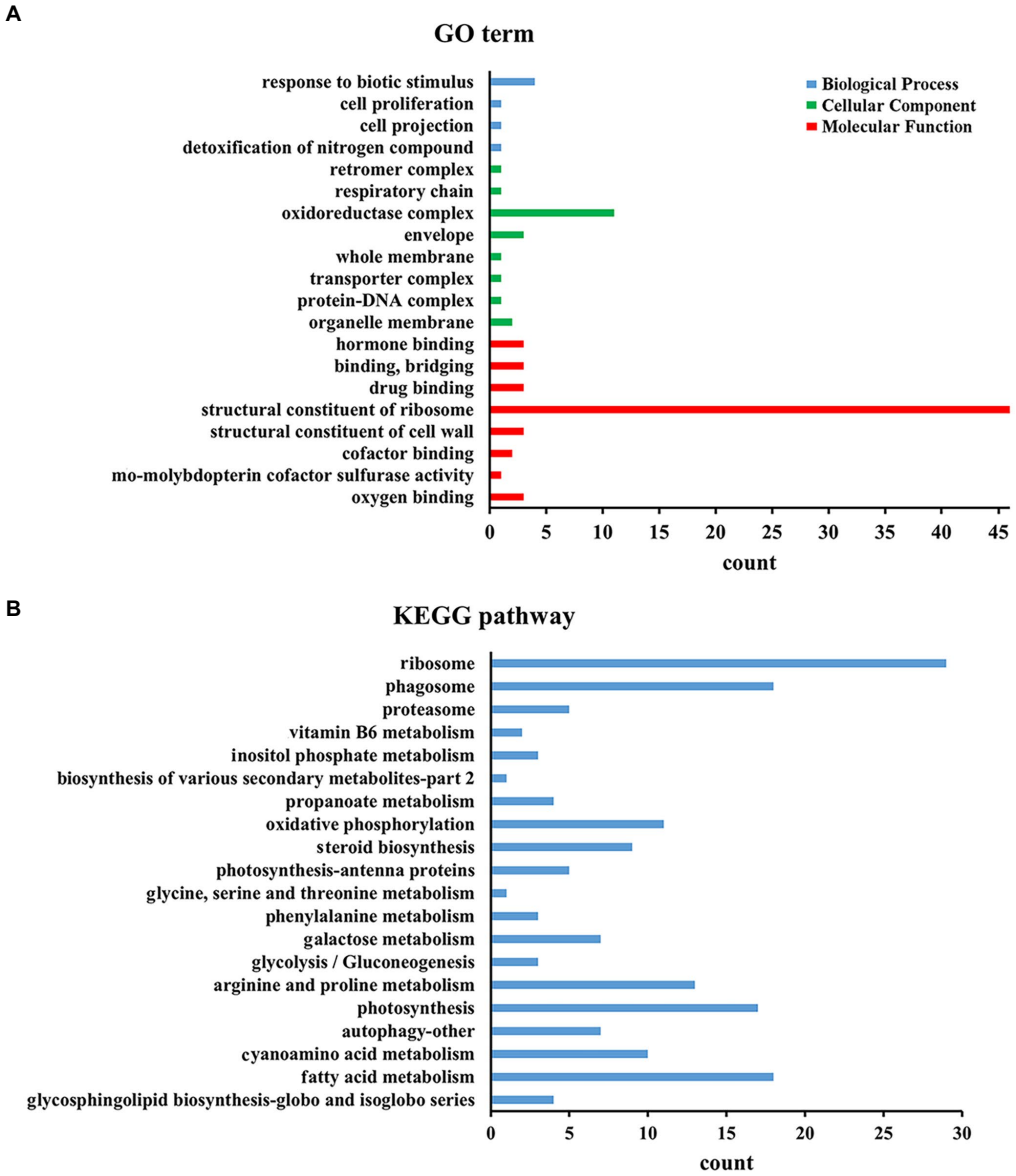
Top KEGG and GO enrichment analyses of all the DEGs. **(A)** Top 20 KEGG enrichment of all the DEGs. **(B)** Top 20 GO enrichment of all the DEGs.

To determine whether DElncRNAs are functionally involved in the Al stress response process, the functions of DElncRNAs in different periods under Al stress were inferred by applying GO category enrichment analysis. According to the results of the GO enrichment analysis of all DElncRNAs, DElncRNAs were mostly enriched in ribosomes in terms of molecular functional category, followed by the oxidoreductase complex in the cellular component category and response to biotic stimulus in the biological process category (Figure 3B). In addition, 0 h vs. 48 h had the most DElncRNAs, among which DElncRNAs were most distributed in binding and catalytic activity of molecular functions, cell and cell part of cellular components, and metabolic process and cellular process of biological processes (Supplementary Table S6).

To understand the metabolism of *M. truncatula* under Al stress, we mapped all DElncRNAs to the KEGG database contents to determine their involved metabolic enrichment pathways. Among them, ribosome, phagosome and fatty acid metabolism were the most abundant KEGG enrichment pathways (Figure 3B). We also observed that the tricarboxylic acid (TCA) cycle at 0 h vs. 48 h involves 21 target genes, mainly enriched in carbon metabolism (ko01200), glyoxylate and dicarboxylate metabolism (ko00630), malate dehydrogenase (ko00026) and other pathways (Supplementary Tables S7, S8).

**Figure 4:**
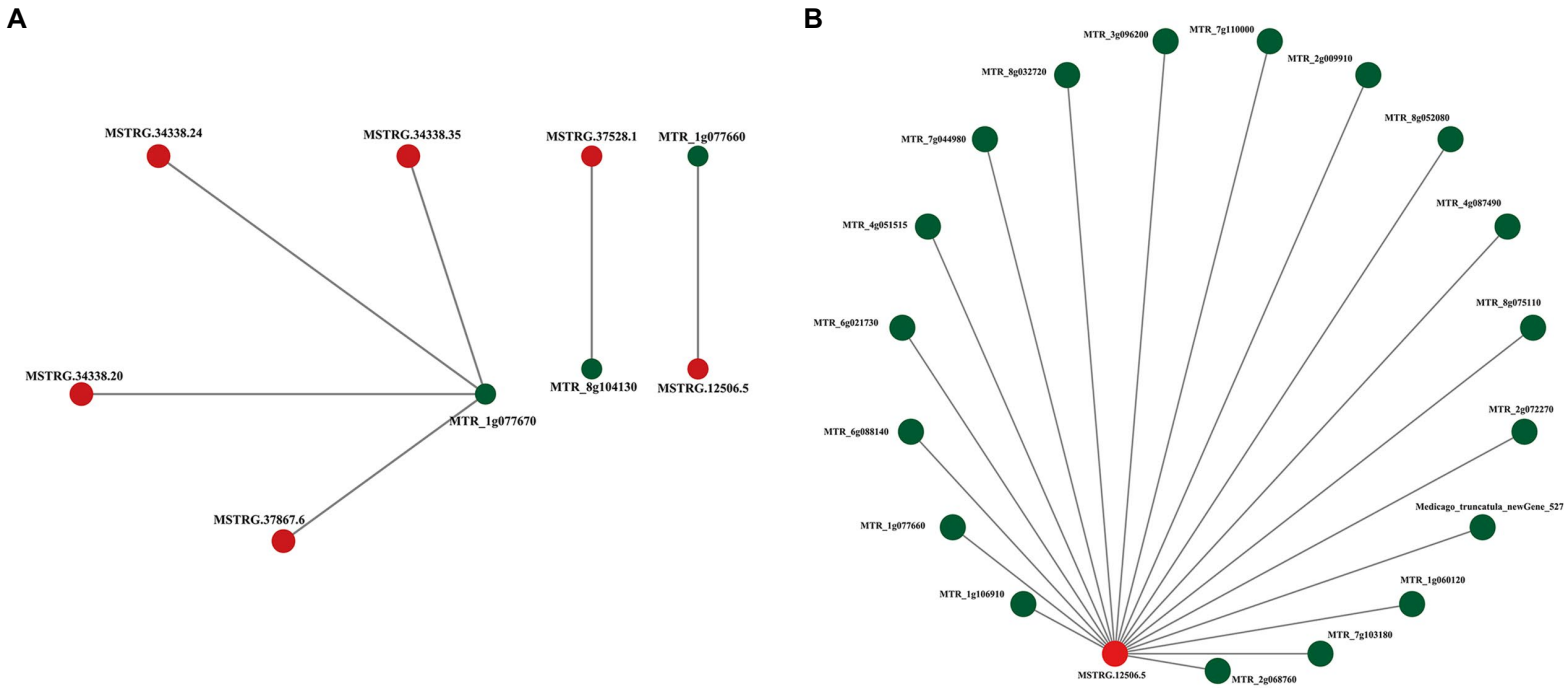
Representatives of predicted interaction networks among ALMT-related lncRNAs and protein-coding RNAs. **(A)** Interaction network of MTR_1g077670. **(B)** Interaction network of MSTRG.12506.5. The lncRNA and mRNA nodes are colored red and green, respectively.

To reveal the relationship between lncRNAs coexpressed and separated by less than 200 kb and protein-coding RNAs, the interaction network of *ALMT*-related mRNA (MTR_1g077670) and lncRNA (MSTRG.12506.5) was constructed using Cytoscape software (Figures 4A,B). The results show that there are both single nodes and complex networks with more than three nodes in the interactive network. For example, the *ALMT*-related mRNA MTR_1g077670 was regulated by 4 lncRNAs, while the lncRNA MSTRG.12506.5 may be involved in the regulation of 18 mRNAs. Thus, the network between these lncRNAs and mRNAs may play important roles in the response to Al stress and malate synthesis.

### Identification of lncRNAs related to the tricarboxylic acid cycle under Al stress

**Figure 5:**
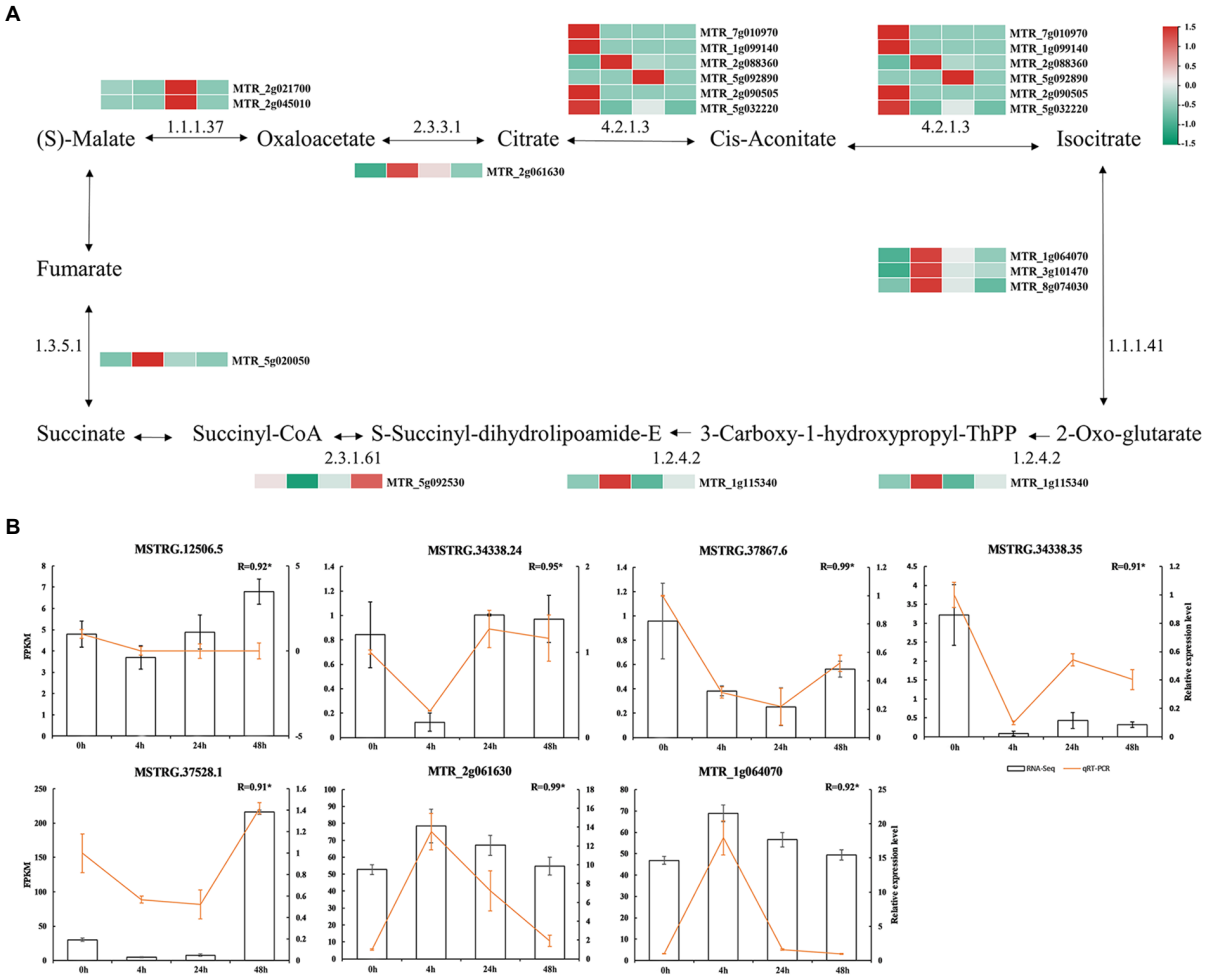
Malate and citric acid metabolic pathways. **(A)** mRNA involved in the regulation of malate and citric acid metabolism. The colored blocks represent the aluminum stress treatment time, 0 h, 4 h, 24 h and 48 h from left to right. **(B)** Validation of lncRNAs. Bars represent RNA-Seq and yellow lines represent qRT-PCR.

At 0 h vs. 48 h, we identified 15 mRNAs targeted by TCA cycle-related lncRNAs (Figure 5A; Supplementary Table S9). Among them, malate dehydrogenase mRNA (MTR_2g021700 and MTR_2g045010) related to malate metabolism was significantly more abundant at 24 h than it was in the other treatments, while citrate synthase mRNA (MTR_2g061630) related to citrate metabolism was significantly more abundant at 4 h than in the other treatments. It was suggested that the synthesis of citrate and malate may be regulated by lncRNAs with different expression patterns in response to Al stress.

To verify the results of RNA-seq, we randomly selected five DElncRNAs in the TCA cycle pathway (MSTRG.12506.5, Al-activated malate transporter 10; MSTRG.34338.24, Al-activated malate transporter 10; MSTRG.34338.35, Al-activated malate transporter 10; malate transporter 10; MSTRG.37528.1, Al-activated malate transporter family protein; MSTRG.37867.6, Al-activated malate transporter 10), and two mRNAs (MTR_2g061630, citrate synthase; and MTR_1g064070, isocitrate dehydrogenase) were measured by qRT–PCR. The expression trends of DElncRNAs were consistent with the results of RNA-seq, indicating the reliability of the expression analysis results (Figure 5B).

### Determination of DElncRNA functions in transgenic yeast

**Figure 6:**
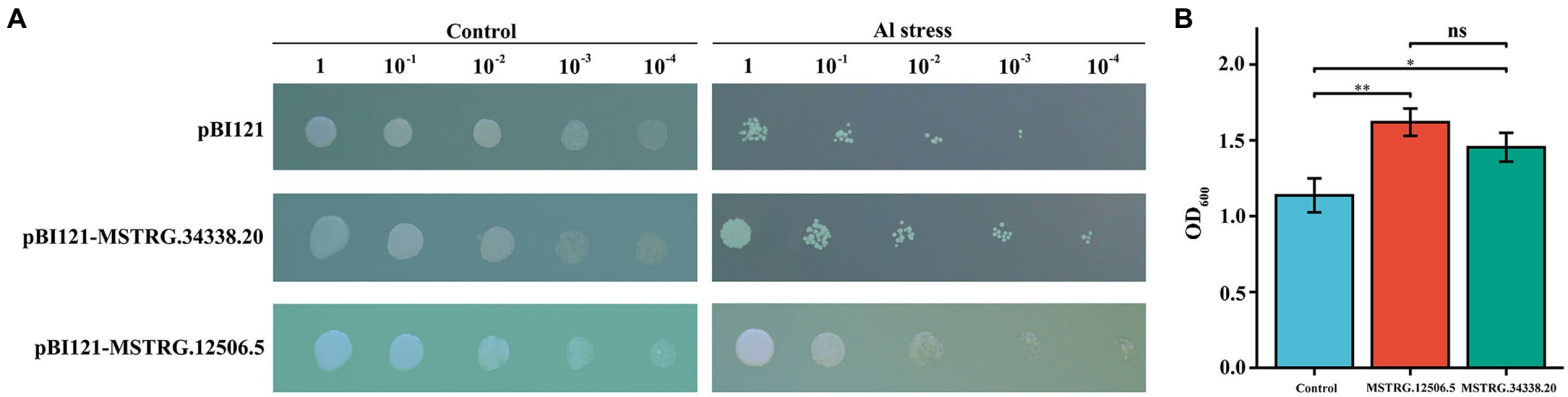
Phenotypic growth assay and concentration assay of transgenic INVSc1 cells under Al stress. **(A)** Aliquots of serially diluted (1, 10^−1^, 10^−2^, 10^−3^, and 10^−4^) cultures on 10 μM Al SC-Ura media with the yeast containing a pBI121 empty vector, pBI121-MSTRG.12506.5, or pBI121-MSTRG.34338.20. **(B)** Yeast growth in SCu/2% (w/v) glucose liquid media supplemented with 10 μM Al.

To further investigate the relationship between the identified DElncRNAs and Al stress, two *ALMT*-related DElncRNAs (MSTRG.12506.5 and MSTRG.34338.20) were overexpressed in the *Saccharomyces cerevisiae* yeast strain INVSc1, and the growth characteristics of the transformants were observed. There was no difference in the growth rates of the yeast transformed with the empty and recombinant plasmids on plates of media without AlCl_3_ (Figure 6A). On plates with media supplemented with 10 μM AlCl_3_, the recombinant plasmid cells showed better growth than empty vector cells, and pBI121-MSTRG.12506.5 and pBI121-MSTRG.34338.20 could still grow at a 10^−4^ dilution, but the pBI121 transgenic yeast cells were barely able to grow at the 10^−4^ dilution (Figure 6A). In the liquid media that included 10 μM AlCl_3_, the OD_600_ of the transgenic yeasts pBI121-MSTRG.12506.5 and pBI121-MSTRG.34338.20 was significantly higher than that of the yeast harboring empty pBI121 vectors (Figure 6B). These results indicated that potential candidate genes with Al stress tolerance can be screened in these DElncRNAs, and the Al tolerance of legumes can be improved by molecular breeding.

## Discussion

### lncRNA is involved in the regulation of Al stress tolerance

Al in acidic soil is one of the main toxic metal ions that inhibits the growth and development of roots and aerial parts of crop species such as *Triticum aestivum*, *Oryza sativa* and *Medicago sativa* and, in severe cases, causes death (Chauhan et al., 2021; Jiang et al., 2022). Plants growing in acidic soils have evolved complex adaptive mechanisms to cope with Al stress. Understanding the molecular mechanisms of the Al stress response is important for Al-tolerant crop plants breeding programs. Studies have shown that ncRNAs play important roles in many important biological processes, among which lncRNAs have received increasing attention in regulating plant responses to biotic and abiotic stresses, such as drought stress in *Zea mays* and *Pyrus* spp. (Zhang et al., 2014; Wang et al., 2018) as well as cold stress and Pi stress in *M. truncatula* (Wang et al., 2017; Zhao et al., 2020). However, lncRNAs associated with Al stress have received little research attention. In this study, we identified 3,284 unique lncRNAs by strand-specific RNA-seq, the number of which was smaller than the number obtained during *M. truncatula* nodule senescence (4,576), salt stress (23,324) and cold stress (24,368) (Wang et al., 2015; Zhao et al., 2020; Yu et al., 2022). These discrepancies may be due to the different tissue sites, or different susceptibilities of *M. truncatula* to different biotic or abiotic stresses (Wang et al., 2015).

### Hormonal signaling-related lncRNAs involved in the Al stress response

Plant hormones can be divided into two categories based on their response to the external environment. One category is “positive growth regulators,” which include hormones such as auxin (IAA), gibberellin (GA) and cytokinin (CK). Another category is “stress hormones,” which include hormones such as abscisic acid (ABA), ethylene (ET) and jasmonic acid (JA) (Ahres et al., 2021). IAAs are a class of hormones that regulate cell division and elongation in plants (Yan et al., 2022). Al inhibits the transport of IAA from plant shoots to root tips. A previous study found that the Al resistance of maize is mainly related to the distal transition zone (DTZ) 1–2 mm from the root tip, and the signaling pathway of the root tip can mediate Al signaling between the DTZ and the elongation zone (EZ) 2.5–5 mm from the apex through basipetal IAA transport, and exogenous indole-3-acetic acid to the EZ site can significantly alleviate the inhibition of root elongation induced by Al stress (Kollmeier et al., 2000). In the present study, after 48 h of Al treatment of *M. truncatula*, a total of 184 lncRNAs regulating plant hormone signal transduction were detected (Supplementary Table S10). Among them, 88 lncRNAs (8 upregulated, 80 downregulated) were related to IAA, and 9 (1 upregulated, 8 downregulated) were related to the synthesis of indole-3-acetic acid. These results suggest that the correlation between lncRNAs and mRNAs may exist in the regulation of IAA and indole-3-acetic acid synthesis and transport in response to Al stress. IAA response factors (ARFs) are transcription factors involved in IAA signaling downstream of TIR1/AFB in the IAA signaling pathway. IAA-regulated root growth inhibition induced by Al stress is mainly mediated by ARFs (Liu et al., 2022). We detected 62 ARF-related lncRNAs regulating 6 mRNAs (Supplementary Table S10). Furthermore, previous studies have shown that the downregulation of small IAA-upregulated RNA (SAUR)-related genes reflects a compensatory mechanism of plants in response to abiotic stresses such as cold, drought, and salt (Stortenbeker and Bemer 2019). In the current study, we identified 40 SAUR-related lncRNAs that regulate 16 mRNAs (Supplementary Table S10), of which 34 lncRNAs and 12 mRNAs were downregulated, suggesting that *M. truncatula* may also employ a similar compensatory mechanism under Al stress.

### Function of the cell wall in Al stress tolerance

In plants, the cell wall is the first barrier against biotic or abiotic stress, and root tip cell walls are considered to be the main sites of Al toxicity and Al rejection (Nagayama et al., 2022). Studies have found that up to 90% of the Al^3+^ absorbed by plants is distributed in the apoplast of the cell, and pectin, the main cell wall polysaccharide of the apoplast, has many carboxyl groups and a strong affinity for Al_3+_ (Schmohl et al., 2000; Liu et al., 2022). This causes excess Al to bind to the cell wall, thereby altering the composition and structure of the cell wall and ultimately disrupting and inhibiting cell wall and root elongation. Polygalacturonase (PG), a type of pectin-digesting enzyme, belongs to glycosyl hydrolase family 28 (GH28) and plays a major role in the degradation of components of the pectin network (Wang et al., 2016; Kim et al., 2019). Overexpression of the *MsPG4* gene in alfalfa can reduce the content of water-soluble pectin and chelator soluble pectin and effectively improve its cell wall extension and Al resistance (Fan et al., 2022). In this study, we detected 11 polygalacturonase-related DElncRNAs regulating 12 mRNAs, of which 4 were upregulated and 8 were downregulated (Supplementary Table S11). These DElncRNAs, which are upregulated under Al stress, may be involved in regulating the hydrolysis of the cell wall pectin network during root growth, reducing Al accumulation in the cell wall and contributing to cell wall elongation, thereby protecting roots from Al-induced elongation inhibition.

### Malate synthesis and citric acid synthesis-related lncRNAs play important roles in the Al stress response

There are two main mechanisms of Al tolerance in plants: one is the exclusion mechanism, in which Al ions are prevented from entering the root tip cells through the apoplastic (cell wall) pathway; the other relies on the intracellular symplastic (cytosolic) pathway for detoxification and the mechanism of vacuolar sequestration (Kochian et al., 2004; Wang et al., 2020). Most monocots and dicots secrete organic acids such as malic acid, citric acid and oxalic acid from the roots to form chelates with Al_3+_ in the rhizosphere, thereby reducing the harm of Al toxicity. This mode is the most typical and effective Al exclusion mechanism (Dissanayaka et al., 2021). In plants, *ALMT* encodes the malic acid transporter, and the multidrug and toxic compound extrusion-related gene *MATE* encodes a citrate transporter. Al stress can significantly increase the expression of *ALMT* and *MATE* and promote the secretion of malic acid and citric acid from roots (Upadhyay et al., 2019; Liu et al., 2022). In *A. thaliana*, Al stress-induced *AtALMT1* gene expression promoted root malate exudation (Magalhaes 2006). In this study, we detected 16 *ALMT* family-associated DElncRNAs involved in the regulation of 3 mRNAs and 3 *MATE* family-associated DElncRNAs involved in the regulation of 3 mRNAs (Supplementary Tables S12, S13). Further research found that STOP1, a C2H2-type transcription factor, plays an important role in plant Al stress tolerance. After sensing Al_3+_ signals, plants highly express STOP1 in their cells, thereby regulating the expression of *ALMT1* and *MATE* (Liu et al., 2022). We detected 8 C2H2-type transcription factor-related DElncRNAs (Supplementary Table S14) involved in the regulation of 1 mRNA, but only 1 DElncRNA was upregulated at 48 h, and its relationship with Al^3+^ signaling remains unknown.

Our validation experiments further demonstrated that DElncRNAs related to *ALMT* families can regulate malate production. Under conditions of 10 μM AlCl_3_, by targeting the lncRNAs MSTRG.12506.5 and MSTRG.34338.20 predicted to target the regulatory mRNAs MTR_1g077660 and MTR_1g077670, we performed transgenic yeast validation experiments. The results showed that growth of the transformants was significantly better than that of the empty vector-transformed controls, indicating that lncRNAs could adapt to Al stress by regulating malate synthesis-related genes and increasing malate secretion.

## Conclusion

We obtained a total of 219.49 Gn of clean sequence data from 12 paired-end library sequences and identified 3,284 lncRNAs. Through GO and KEGG enrichment of lncRNA targets in response to Al stress, we found that these lncRNAs are involved in the regulation of plant hormone signal transduction and the TCA cycle. We further demonstrated that the lncRNAs MSTRG.12506.5 and MSTRG.34338.20 are involved in the regulation of the malate synthesis network. These results provide new insights into the function of lncRNAs in response to Al stress.

## Data availability statement

The datasets presented in this study can be found in online repositories. The names of the repository/repositories and accession number(s) can be found at: https://www.ncbi.nlm.nih.gov/, PRJNA868891.

## Author contributions

RD and CC conceived the experiment. QG, ZY, FY, and SW carried it out. QG and ZY analyzed the data. RD and QG wrote the paper. All authors contributed to the article and approved the submitted version.

## Funding

This research was supported by the National Natural Science Foundation of China (32060392) and Support by Guizhou Province Science and Technology Projects (Qian Ke He Zhi Cheng [2020]1Y074).

## Conflict of interest

The authors declare that the research was conducted in the absence of any commercial or financial relationships that could be construed as a potential conflict of interest.

## Publisher’s note

All claims expressed in this article are solely those of the authors and do not necessarily represent those of their affiliated organizations, or those of the publisher, the editors and the reviewers. Any product that may be evaluated in this article, or claim that may be made by its manufacturer, is not guaranteed or endorsed by the publisher.

## References

[ref1] Ahres M. Pálmai T. Gierczik K. Dobrev P. Vanková R. Galiba G. (2021). The impact of far-red light supplementation on hormonal responses to cold acclimation in barley. Biomol. Ther. 11:450. doi: 10.3390/biom11030450, PMID: 33802867 PMC8002655

[ref2] Anders S. Huber W. (2010). Differential expression analysis for sequence count data. Genome Biol. 11:R106. doi: 10.1186/gb-2010-11-10-r106, PMID: 20979621 PMC3218662

[ref3] Bugbee B. (2004). Nutrient management in recirculating hydroponic culture. Acta Hortic. 648, 99–112. doi: 10.17660/ActaHortic.2004.648.12

[ref4] Chauhan D. K. Yadav V. Vaculík M. Gassmann W. Pike S. Arif N.. (2021). Aluminum toxicity and aluminum stress-induced physiological tolerance responses in higher plants. Crit. Rev. Biotechnol. 41, 715–730. doi: 10.1080/07388551.2021.1874282, PMID: 33866893

[ref5] Chen L. Shi S. Jiang N. Khanzada H. Wassan G. M. Zhu C.. (2018). Genome-wide analysis of long non-coding RNAs affecting roots development at an early stage in the rice response to cadmium stress. BMC Genomics 19, 460–410. doi: 10.1186/s12864-018-4807-6, PMID: 29902991 PMC6002989

[ref6] Csorba T. Questa J. I. Sun Q. Dean C. (2014). Antisense COOLAIR mediates the coordinated switching of chromatin states at FLC during vernalization. Proc. Natl. Acad. Sci. U. S. A. 111, 16160–16165. doi: 10.1016/S1369-5266(99)80053-3, PMID: 25349421 PMC4234544

[ref7] Cui J. Jiang N. Hou X. Wu S. Zhang Q. Meng J.. (2020). Genome-wide identification of lncRNAs and analysis of ceRNA networks during tomato resistance to Phytophthora infestans. Phytopathology 110, 456–464. doi: 10.1094/PHYTO-04-19-0137-R, PMID: 31448997

[ref8] Dissanayaka D. Ghahremani M. Siebers M. Wasaki J. Plaxton W. C. (2021). Recent insights into the metabolic adaptations of phosphorus-deprived plants. J. Exp. Bot. 72, 199–223. doi: 10.1093/jxb/eraa482, PMID: 33211873

[ref9] Fahlgren N. Carrington J. C. (2010). miRNA target prediction in plants. Methods Mol. Biol. 592, 51–57. doi: 10.1007/978-1-60327-005-2_4 19802588

[ref001] Fan N. Wen W. Gao L. Lv A. Su L. Zhou P.. (2022). MsPG4-mediated hydrolysis of pectins increases the cell wall extensibility and aluminum resistance of alfalfa. Plant Soil 1–15. doi: 10.1007/s11104-022-05431-3

[ref10] Frazee A. C. Pertea G. Jaffe A. E. Langmead B. Salzberg S. L. Leek J. T. (2015). Ballgown bridges the gap between transcriptome assembly and expression analysis. Nat. Biotechnol. 33, 243–246. doi: 10.1038/nbt.3172, PMID: 25748911 PMC4792117

[ref002] Giulietti A. Overbergh L. Valckx D. Decallonne B. Bouillon R. Mathieu C. (2001). An overview of real-time quantitative PCR: applications to quantify cytokine gene expression. Methods 25, 386–401. doi: 10.1006/meth.2001.1261 11846608

[ref11] Jiang X. Li W. Han M. Chen G. Wu J. Lai S. Y.. (2022). Aluminum-tolerant, growth-promoting endophytic bacteria as contributors in promoting tea plant growth and alleviating aluminum stress. Tree Physiol. 42, 1043–1058. doi: 10.1093/treephys/tpab159, PMID: 34850946 PMC9092644

[ref12] Kawai S. Hashimoto W. Murata K. (2010). Transformation of Saccharomyces cerevisiae and other fungi: methods and possible underlying mechanism. Bioengineered 1, 395–403. doi: 10.4161/bbug.1.6.13257, PMID: 21468206 PMC3056089

[ref004] Kim J. Chun J. P. Tucker M. L. (2019). Transcriptional regulation of abscission zones. Plants. 8:154. doi: 10.3390/plants8060154 PMC6631628 31174352

[ref13] Kochian L. V. Hoekenga O. A. Pineros M. A. (2004). How do crop plants tolerate acid soils? Mechanisms of aluminum tolerance and phosphorous efficiency. Annu. Rev. Plant Biol. 55, 459–493. doi: 10.1146/annurev.arplant.55.031903.141655, PMID: 15377228

[ref14] Kollmeier M. Felle H. H. Horst W. J. (2000). Genotypical differences in aluminum resistance of maize are expressed in the distal part of the transition zone. Is reduced basipetal auxin flow involved in inhibition of root elongation by aluminum? Plant Physiol. 122, 945–956. doi: 10.1104/pp.122.3.945, PMID: 10712559 PMC58931

[ref15] Kong L. Zhang Y. Ye Z. Liu X. Zhao S. Wei L.. (2007). CPC: assess the protein-coding potential of transcripts using sequence features and support vector machine. Nucleic Acids Res. 35, W345–W349. doi: 10.1093/nar/gkm391, PMID: 17631615 PMC1933232

[ref16] Li C. X. Yan J. Y. Ren J. Y. Sun L. Xu C. Li G. X.. (2020). A WRKY transcription factor confers aluminum tolerance via regulation of cell wall modifying genes. J. Integr. Plant Biol. 62, 1176–1192. doi: 10.1111/jipb.12888, PMID: 31729146

[ref005] Liu H. Heckman J. R. Murphy J. A. (1995). Screening Kentucky bluegrass for aluminum tolerance. J. Plant Nutr. 18, 1797–1814. doi: 10.1080/01904169509365024

[ref17] Liu H. Zhu R. Shu K. Lv W. Wang S. Wang C. L. (2022). Aluminum stress signaling, response, and adaptive mechanisms in plants. Plant Signal. Behav. 17:2057060. doi: 10.1080/15592324.2022.2057060, PMID: 35467484 PMC9045826

[ref18] Liu W. Zhang Z. Chen S. Ma L. Wang H. Dong R.. (2016). Global transcriptome profiling analysis reveals insight into saliva-responsive genes in alfalfa. Plant Cell Rep. 35, 561–571. doi: 10.1007/s00299-015-1903-9, PMID: 26645698

[ref19] Lou H. Q. Gong Y. L. Fan W. Xu J. M. Liu Y. Cao M. J.. (2016). A formate dehydrogenase confers tolerance to aluminum and low pH. Plant Physiol. 171, 294–305. doi: 10.1104/pp.16.01105, PMID: 27021188 PMC4854670

[ref20] Magalhaes J. V. (2006). Aluminum tolerance genes are conserved between monocots and dicots. Proc. Natl. Acad. Sci. U. S. A. 103, 9749–9750. doi: 10.1073/pnas.0603957103, PMID: 16785425 PMC1502523

[ref21] Mao X. Cai T. Olyarchuk J. G. Wei L. (2005). Automated genome annotation and pathway identification using the KEGG Orthology (KO) as a controlled vocabulary. Bioinformatics 21, 3787–3793. doi: 10.1093/bioinformatics/bti430, PMID: 15817693

[ref006] Nagayama T. Tatsumi A. Nakamura A. Yamaji N. Satoh S. Furukawa J.. (2022). Effects of polygalacturonase overexpression on pectin distribution in the elongation zones of roots under aluminium stress. AoB Plants 14:plac003. doi: 10.1093/aobpla/plac003 35356145 PMC8963292

[ref22] Pertea M. Pertea G. M. Antonescu C. M. Chang T. Mendell J. T. Salzberg S. L.. (2015). StringTie enables improved reconstruction of a transcriptome from RNA-seq reads. Nat. Biotechnol. 33, 290–295. doi: 10.1038/nbt.3122, PMID: 25690850 PMC4643835

[ref23] Ponting C. P. Oliver P. L. Reik W. (2009). Evolution and functions of long noncoding RNAs. Cells 136, 629–641. doi: 10.1016/j.cell.2009.02.006 19239885

[ref24] Poot-Poot W. Hernandez-Sotomayor S. M. (2011). Aluminum stress and its role in the phospholipid signaling pathway in plants and possible biotechnological applications. IUBMB Life 63, 864–872. doi: 10.1002/iub.550, PMID: 21905199

[ref007] Schmohl N. Pilling J. Fisahn J. Horst W. J. (2000). Pectin methylesterase modulates aluminium sensitivity in Zea mays and Solanum tuberosum. Physiol. Plant. 109, 419–427. doi: 10.1034/j.1399-3054.2000.100408.x

[ref25] Stortenbeker N. Bemer M. (2019). The SAUR gene family: the plant's toolbox for adaptation of growth and development. J. Exp. Bot. 70, 17–27. doi: 10.1093/jxb/ery332, PMID: 30239806

[ref26] Sun L. Luo H. Bu D. Zhao G. Yu K. Zhang C.. (2013). Utilizing sequence intrinsic composition to classify protein-coding and long non-coding transcripts. Nucleic Acids Res. 41:e166. doi: 10.1093/nar/gkt646, PMID: 23892401 PMC3783192

[ref27] Tokizawa M. Enomoto T. Ito H. Wu L. Kobayashi Y. Mora-Macías J.. (2021). High affinity promoter binding of STOP1 is essential for early expression of novel aluminum-induced resistance genes GDH1 and GDH2 in Arabidopsis. J. Exp. Bot. 72, 2769–2789. doi: 10.1093/jxb/erab031, PMID: 33481007

[ref28] Upadhyay N. Kar D. Deepak Mahajan B. Nanda S. Rahiman R. Panchakshari N.. (2019). The multitasking abilities of MATE transporters in plants. J. Exp. Bot. 70, 4643–4656. doi: 10.1093/jxb/erz246, PMID: 31106838

[ref29] Von Uexküll H. R. Mutert E. (1995). Global extent, development and economic impact of acid soils. Plant Soil 171, 1–15. doi: 10.1007/BF00009558

[ref003] Wang F. Sun X. Shi X. Zhai H. Tian C. Kong F.. (2016). A global analysis of the polygalacturonase gene family in soybean (*Glycine max*). PloS One 11:e0163012. doi: 10.1371/journal.pone.0163012 27657691 PMC5033254

[ref30] Wang A. Hu J. Gao C. Chen G. Wang B.. (2019). Genome-wide analysis of long non-coding RNAs unveils the regulatory roles in the heat tolerance of Chinese cabbage (Brassica rapa ssp. chinensis). Sci. Rep. 9:5002. doi: 10.1038/s41598-019-41428-2, PMID: 30899041 PMC6428831

[ref31] Wang J. Lin J. Kan J. Wang H. Li X. Yang Q.. (2018). Genome-wide identification and functional prediction of novel drought-responsive lncRNAs in Pyrus betulifolia. Genes 9:311. doi: 10.3390/genes9060311, PMID: 29925818 PMC6027255

[ref32] Wang L. Park H. J. Dasari S. Wang S. Kocher J. Li W. (2013). CPAT: coding-potential assessment tool using an alignment-free logistic regression model. Nucleic Acids Res. 41:e74. doi: 10.1093/nar/gkt006, PMID: 23335781 PMC3616698

[ref33] Wang T. Liu M. Zhao M. Chen R. Zhang W. (2015). Identification and characterization of long non-coding RNAs involved in osmotic and salt stress in Medicago truncatula using genome-wide high-throughput sequencing. BMC Plant Biol. 15, 131–113. doi: 10.1186/s12870-015-0530-5, PMID: 26048392 PMC4457090

[ref34] Wang T. Zhao M. Zhang X. Liu M. Yang C. Chen Y.. (2017). Novel phosphate deficiency-responsive long non-coding RNAs in the legume model plant Medicago truncatula. J. Exp. Bot. 68, 5937–5948. doi: 10.1093/jxb/erx384, PMID: 29165588 PMC5854128

[ref35] Wang Y. Yu W. Cao Y. Cai Y. Lyi S. M. Wu W.. (2020). An exclusion mechanism is epistatic to an internal detoxification mechanism in aluminum resistance in Arabidopsis. BMC Plant Biol. 20, 122–112. doi: 10.1186/s12870-020-02338-y, PMID: 32188405 PMC7079475

[ref37] Yan L. Riaz M. Liu J. Yu M. Cuncang J. (2022). The aluminum tolerance and detoxification mechanisms in plants; recent advances and prospects. Crit. Rev. Environ. Sci. Technol. 52, 1491–1527. doi: 10.1093/jxb/erj131

[ref38] Yu L. Huang T. Qi X. Yu J. Wu T. Luo Z.. (2022). Genome-wide analysis of long non-coding RNAs involved in nodule senescence in medicago truncatula. Front. Plant Sci. 13:917840. doi: 10.3389/fpls.2022.917840, PMID: 35707611 PMC9189404

[ref39] Zhan J. Li W. He H. Li C. He L. (2014). Mitochondrial alterations during Al-induced PCD in peanut root tips. PLANT PHYSIOL BIOCH 75, 105–113. doi: 10.1016/j.plaphy.2013.12.010, PMID: 24398246

[ref40] Zhang W. Han Z. Guo Q. Liu Y. Zheng Y. Wu F.. (2014). Identification of maize long non-coding RNAs responsive to drought stress. PLoS One 9:e98958. doi: 10.1371/journal.pone.0098958, PMID: 24892290 PMC4044008

[ref41] Zhao M. Wang T. Sun T. Yu X. Tian R. Zhang W. H.. (2020). Identification of tissue-specific and cold-responsive lncRNAs in Medicago truncatula by high-throughput RNA sequencing. BMC Plant Biol. 20:99. doi: 10.1186/s12870-020-2301-1, PMID: 32138663 PMC7059299

[ref42] Zhu B. Yang Y. Li R. Fu D. Wen L. Luo Y.. (2015). RNA sequencing and functional analysis implicate the regulatory role of long non-coding RNAs in tomato fruit ripening. J. Exp. Bot. 66, 4483–4495. doi: 10.1093/jxb/erv203, PMID: 25948705 PMC4507755

