# Supplementary figures and images for: Identification and characterization of long noncoding RNAs involved in the aluminum stress response in Medicago truncatula via genome-wide analysis
Source: Front Plant Sci. 2022 Sep 23;13:1017869. doi: 10.3389/fpls.2022.1017869 (PMC9541535; doi:10.3389/fpls.2022.1017869)

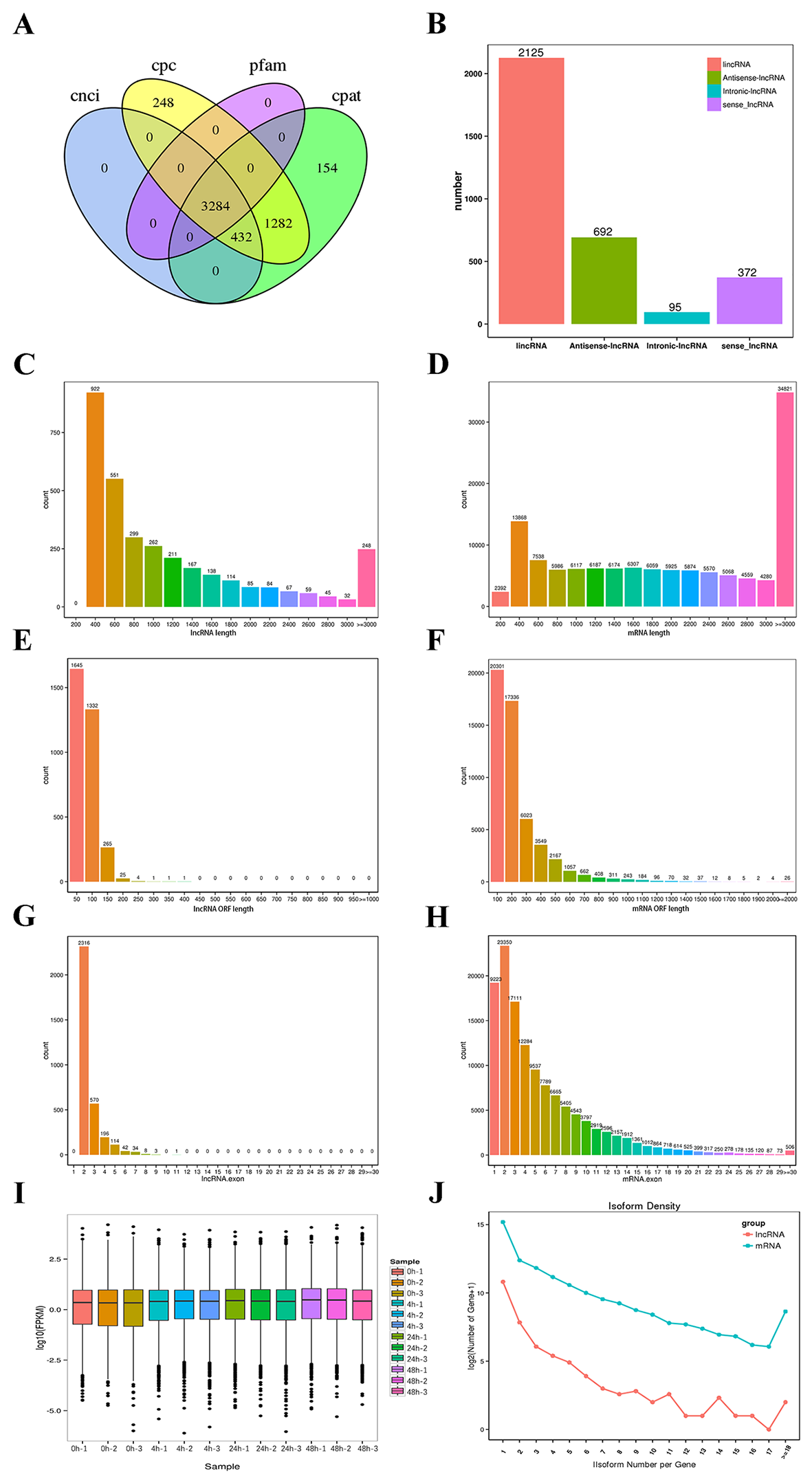

Supplement: Supplementary file 1 [file Image_1.TIF]
